# Plant extracts: a potential approach to combating multidrug-resistant bacteria

**DOI:** 10.17179/excli2026-9282

**Published:** 2026-02-27

**Authors:** Sun Sik Kong, Chang Ha Park

**Affiliations:** 1 College of General Education, Namseoul University, 91 Daehak-ro, Seonghwan-eup, Seobuk-gu, Cheonan-si, Chungcheongnam-do 31020, Republic of Korea; 2 Department of Smart Farm, Namseoul University, 91 Daehak-ro, Seonghwan-eup, Seobuk-gu, Cheonan-si, Chungcheongnam-do 31020, Republic of Korea

## ⁯⁯

Multidrug-resistant (MDR) bacteria are defined as strains susceptible to at least one agent in three or more antimicrobial categories, a classification that has enabled standardized global surveillance and comparative risk assessment (Magiorakos et al., 2012[8]). The WHO Bacterial Priority Pathogens List (WHO, 2024[15]) identified 24 antibiotic-resistant pathogens across 15 bacterial families, including Gram-negative bacteria resistant to last-resort antibiotics, drug-resistant *Mycobacterium tuberculosis*, and other high-burden pathogens such as *Salmonella* spp., *Shigella* spp., *Neisseria gonorrhoeae*, *Pseudomonas aeruginosa*, and *Staphylococcus aureus* (WHO, 2024[15]). Regional surveillance data from the European Centre for Disease Prevention and Control **(**ECDC) have further demonstrated an increasing incidence of bloodstream and healthcare-associated infections caused by resistant organisms across Europe (ECDC, 2024[2]). Collectively, these findings confirm that multidrug resistance represents a widespread and escalating global threat, rather than a pathogen-specific problem.

Most conventional antibiotics inhibit a single essential bacterial function, such as cell wall biosynthesis, protein translation, or DNA replication. Although this approach is highly potent, it facilitates rapid evolutionary escape through point mutations, target modification, enzymatic inactivation, efflux pump activation, or reduced membrane permeability (Wright, 2011[17]; Munita and Arias, 2016[10]). Simultaneously, the antibiotic development pipeline has stagnated. Analyses of the global antibacterial pipeline have consistently shown that the rate of novel antibiotic approval is insufficient to counterbalance the accelerated emergence of drug resistance (WHO, 2022[14], 2024[16]).

In particular, biofilm-associated infections pose major clinical challenges. Bacteria embedded within a self-produced extracellular matrix exhibit markedly increased tolerance to antimicrobial agents compared with planktonic cells (Costerton et al., 1999[1]; Mah, 2012[9]; Uruén et al., 2020[13]). Biofilms restrict antibiotic diffusion, promote metabolic dormancy and persister cell formation, and facilitate horizontal gene transfer, collectively enabling persistence and recurrent infection (Uruén et al., 2020[13]; Flemming et al., 2016[3]; Niu et al., 2024[11]). Consequently, even newly developed antibiotics may fail if the biofilm formation is not explicitly targeted (Mah, 2012[9]; Uruén et al., 2020[13]).

Plants have evolved complex innate immune systems to counter microbial attack. Pathogen-associated molecular patterns that activate coordinated hormonal and transcriptional responses, including signaling pathways for salicylic acid, jasmonic acid, and ethylene, have been recognized. These result in the production of antimicrobial proteins and diverse secondary metabolites (Zhang et al., 2025[18]; Kumar et al., 2023[7]). The metabolites, including phenolics, flavonoids, terpenoids, and alkaloids, function as broad-spectrum defense molecules and frequently exhibit multi-target modes of action (Kumar et al., 2023[7]).

Plant-derived metabolites often exert polypharmacological effects, simultaneously disrupting bacterial membranes, inhibiting enzymatic and nucleic-acid-based processes, inducing oxidative stress, interfering with quorum sensing, suppressing efflux pumps, and attenuating biofilm formation (see Supplementary information, Table 1) (Zhang et al., 2025[18]; Kumar et al., 2023[7]; Othman et al., 2019[12]; Khare et al., 2021[6]; Keita et al., 2022[5]). Such multi-target pressure reduces the likelihood of rapid resistance developing from single genetic alterations. Furthermore, previous studies have explained synergistic interactions between plant-derived compounds and conventional antibiotics that restore antibiotic susceptibility in resistant strains (see Supplementary information, Table 2) (Othman et al., 2019[12]; Khare et al., 2021[6]).

While phytochemicals possess multi-target antimicrobial activity, the clinical translation of plant extracts faces challenges related to standardization, reproducibility, pharmacokinetics, bioavailability, safety, and regulatory approval (Jubair et al., 2021[4]; Uruén et al., 2020[13]). In particular, the uniformity in plant cultivation should be investigated since environmental factors, such as soil composition, climate, and harvesting time, directly influence the profile of secondary metabolites. The chemical complexity of plant extracts complicates batch consistency and mechanism-of-action attribution, necessitating rigorous experimental validation using standardized synergy assays and *in vivo* models. While selective for bacterial targets, high doses of plant extracts may exhibit cytotoxicity to mammalian cells or induce allergic reactions. Therefore, long-term toxicity studies are required to establish safety. Furthermore, future work should focus on the design of phytochemical and antibiotic combinations and advanced delivery systems (e.g., nanoformulations, localized hydrogels) to enhance stability, target-site exposure, and synergy while minimizing host toxicity. Collectively, the integration of plant-derived compounds as adjuncts to existing antibiotics or components of novel antimicrobial formulations should be actively explored as a realistic and potentially high-impact strategy against MDR infections.

The antibiotic era initiated by penicillin dramatically reduced morbidity and mortality from bacterial infections. However, its impact is increasingly undermined by the global spread of MDR bacteria and the persistence of biofilm-associated infections (Magiorakos et al., 2012[8]; ECDC, 2024[2]; WHO 2024[15]). Surveillance data from the WHO and ECDC demonstrate that resistance to standard antibiotics now affects a substantial proportion of key bacterial pathogens at regional and global scales. Plant-derived secondary metabolites are biologically plausible and mechanistically diverse reservoirs of antimicrobial agents. Their multi-target activity, antibiofilm properties, and capacity to potentiate conventional antibiotics position them as promising complementary tools in the broader effort to prevent, treat, and contain antimicrobial resistance. Here, this study highlights the rationale for systematically advancing plant-derived antibacterial strategies as adjuncts to current antibiotic therapy.

## Declaration

### Conflict of interest

The authors declare no conflict of interest.

### Artificial Intelligence (AI) - assisted technology

The authors used an LLM exclusively for improving English language clarity and readability in the manuscript preparation.

## Supplementary Material

Supplementary information
