# Supplementary material for: Plant extracts: a potential approach to combating multidrug-resistant bacteria
Source: EXCLI J. 2026 Feb 27;25:372–4. doi: 10.17179/excli2026-9282 (PMC13077163; doi:10.17179/excli2026-9282)
Supplement: Supplementary information [file EXCLI-25-372-s-001.pdf]

*EXCLI Journal 2026;25:372-374 – ISSN 1611-2156*
*Received: January 15, 2026, accepted: February 12, 2026, published: February 27, 2026*

## Supplementary information to:

# Letter to the editor:

# PLANT EXTRACTS: A POTENTIAL APPROACH TO COMBATING MULTIDRUG-RESISTANT BACTERIA

Sun Sik Kong[1], Chang Ha Park[2,*]

[1] College of General Education, Namseoul University, 91 Daehak-ro, Seonghwan-eup, Seobuk-gu, Cheonan-si, Chungcheongnam-do 31020, Republic of Korea
[2] Department of Smart Farm, Namseoul University, 91 Daehak-ro, Seonghwan-eup, Seobuk-gu, Cheonan-si, Chungcheongnam-do 31020, Republic of Korea

* **Corresponding author:** Chang Ha Park, Department of Smart Farm, Namseoul University, 91 Daehak-ro, Seonghwan-eup, Seobuk-gu, Cheonan-si, Chungcheongnam-do 31020, Republic of Korea, Tel.: +82-41-580-3254, E-mail: parkch@nsu.ac.kr

https://dx.doi.org/10.17179/excli2025-9282

This is an Open Access article distributed under the terms of the Creative Commons Attribution License (https://creativecommons.org/licenses/by/4.0/).

**Supplementary Table 1:** Antibacterial activities and synergistic effects of phytochemicals against MDR pathogens

| No | Compounds | Class | Strains | Effects | Reference |
|---|---|---|---|---|---|
| **1** | Xanthohumol, desmethylxanthohumol, lupulone | Phenolics | MRSA T28.1 | Anti-biofilm activity | Bocquet et al., 2019 |
| **2** | 1,8-Cineole | Monoterpenoid | Clinical MRSA | Anti-biofilm activity | Merghni et al., 2018 |
| **3** | Baicalin | Flavone | *S. aureus* | Anti-biofilm activity | Zhang et al., 2020 |
| **4** | 4-phenyl coumarin | Coumarin | MRSA ATCC 33591 | Anti-biofilm activity and anti-adherence activity | da Cunha et al., 2020 |
| **5** | Emodin | Anthraquinone | MRSA252 | MRSA cell wall disruption | Cao et al., 2015 |
| **6** | Artonin E | Xanthones | MRSA BAA-1720 | MRSA membrane disruption | Zajmi et al., 2015 |
| **7** | Oxyresveratrol | Phenolics | Clinically isolated strains and ATCC 33591 | Structural alterations in bacterial septa formation | Joung et al., 2016b |
| **8** | reserpine, quinine, piperine, harmaline | Alkaloids | MRSA ATCC 25923/35931 | Efflux pump inhibitory activity | Mohtar et al., 2009 |
| **9** | Chelerythrine | Alkaloid | MRSA RN4220 | Inhibition of multidrug efflux pumps | Gibbons et al., 2003 |

EXCLI Journal 2026;25:372-374 – ISSN 1611-2156
Received: January 15, 2026, accepted: February 12, 2026, published: February 27, 2026

| No | Compounds | Class | Strains | Effects | Reference |
|---|---|---|---|---|---|
| 10 | Ursolic acid | Triterpe-noid | MRSA ATCC 33591 | Inhibition of PBP2a uprotein by ursolic acid with oxacillin | Zhou et al., 2017 |
| 11 | Sanguisorbi-genin | Triterpe-noid | MRSA ATCC 33591 | Downregulation of penicillin-binding protein 2a and mecA genes | Wang et al., 2021 |
| 12 | Shikonin | Naphtho-quinone | MRSA ATCC 33591 | Anti-MRSA activity via ABC transporter modulation and peptidoglycan binding | Lee et al., 2015 |
| 13 | Cajanin Stilbene Acid | Stilbene | VRE ATCC 700802 | Abnormal Energy Metabolism | Tan et al., 2020 |
| 14 | 6, 6'-dihydroxy-thiobinuphari-dine | Alkaloid | VRE FN-1, ATCC 51299 | Downregulation of vanA gene. | Okamura et al., 2015 |

**Supplementary Table 2:** Antibacterial activities and synergistic effects of phytochemicals against MRSA and VRE

| No | Compounds | Class | Strains | Synergy effect | Reference |
|---|---|---|---|---|---|
| 1 | Brazilin | Catechols | Clinical MRSA | Aminoglycosides | Zuo et al., 2014 |
| 2 | Sophorafla-vanone G | Flavanone | MRSA ATCC 33591 | Ampicillin | Cha et al., 2009 |
| 3 | Rhein | Anthraqui-none glyco-side | Clinical MRSA | Ampicillin | Joung et al., 2012 |
| 4 | Luteolin | Flavones | Clinical MRSA | Ampicillin/Oxacillin | Joung et al., 2016a |
| 5 | Baicalin | Flavonoid | MRSA NCTC 11940 | Benzylpenicillin, methicillin, ampicillin, cefotaxime | Liu et al., 2000 |
| 6 | Clerodane | Diterpenoids | MRSA-ST2071 | Ciprofloxacin, norfloxacin, ofloxacin | Gupta et al., 2016 |
| 7 | Chrysoeriol | Flavonoids | Clinical MRSA | Ciprofloxacin, oxacillin | Lan et al., 2021 |
| 8 | Diosmetin | Flavonoids | MRSA RN4220 | Erythromycin | Chan et al., 2015 |
| 9 | Salvianolate | Coumaric | Clinical MRSA | Fosfomycin, erythromycin, piperacillin-tazobactam, clindamycin | Cheng et al., 2013 |
| 10 | Carnosic acid | Diterpenoids | MRSA ATCC 29213 | Gentamicin | Vázquez et al., 2016 |
| 11 | Galangin | Flavonoid | Clinical MRSA | Gentamicin | Lee et al., 2008 |
| 12 | Fremontone | Isoflavone | MRSA ATCC 1708 | Methicillin | Kumarihamy et al., 2022 |
| 13 | Theasinsensin A | Polyphenol flavonoid | Clinical MRSA | Oxacillin | Hatano et al. 2003 |
| 14 | Berberine | Alkaloids | Clinical MRSA | Oxacillin | Yu et al., 2005 |

| No | Compounds | Class | Strains | Synergy effect | Reference |
|---|---|---|---|---|---|
| **15** | Silibin | Flavonoid | MRSA ATCC 33591 | Oxacillin, ampicillin | Kang et al., 2011 |
| **16** | Allicin | Thiosulfonates | MRSA strain | Silver nanoparticles | Sharifi-Rad et al., 2014 |
| **17** | Artocarpin | Flavone | DMST 20654 | tetracycline, ampicillin, norfloxacin | Septama and Panichayupakaranant, 2016 |
| **18** | Sophoraflavanone G | Flavanone | Clinical MRSA | Vancomycin | Sakagami et al., 1998 |
| **19** | Rosmarinic acid | Polyphenol | Clinical MRSA | Vancomycin | Ekambaram et al., 2016 |
| **20** | Stigmasterol / β-caryophyllene oxide | Terpenoids | MRSA ATCC 33592 | β-lactam antibiotics | Alagasamy et al., 2021 |

### *Conflict of interest*

The authors declare no conflict of interest.

### *Artificial Intelligence (AI) – assisted technology*

The authors used an LLM exclusively for improving English language clarity and readability in the manuscript preparation.

## REFERENCES

Alagasamy SV, Ramanathan S, Chear NJY, Tan WN, Ramachandram DS, Ching-Ga AFT, et al. The potentiation of beta-lactam and anti-bacterial activities of lipophilic constituents from Mesua ferrae leaves against methicillin-resistant Staphylococcus aureus. J Complement Integr Med. 2021;18(2):339–345.

Bocquet L, Sahpaz S, Bonneau N, Beaufay C, Mahieux S, Samaillie J, et al. Phenolic compounds from Humulus lupulus as natural antimicrobial products: new weapons in the fight against methicillin resistant Staphylococcus aureus, Leishmania mexicana and Trypanosoma brucei strains. Molecules. 2019;24(6):1024.

Cao F, Peng W, Li X, Liu M, Li B, Qin R, et al. Emodin is identified as the active component of ether extracts from Rhizoma Polygoni Cuspidati, for anti-MRSA activity. Can J Physiol Pharmacol. 2015;93(6):485–493.

Cha JD, Moon SE, Kim JY, Jung EK, Lee YS. Antibacterial activity of sophoraflavanone G isolated from the roots of Sophora flavescens against methicillin-resistant Staphylococcus aureus. Phytother Res. 2009;23(9):1326–1331.

Chan BC, Han XQ, Lui SL, Wong CW, Wang TB, Cheung DW, et al. Combating against methicillin-resistant Staphylococcus aureus - two fatty acids from Purslane (Portulaca oleracea L.) exhibit synergistic effects with erythromycin. J Pharm Pharmacol. 2015;67(1):107–116.

Cheng C, Liu Y, Song H, Pan L, Li J, Qin Y, et al. Marinopyrrole derivatives as potential antibiotic agents against methicillin-resistant Staphylococcus aureus (II). Marine Drugs. 2013;11(8):2927–2948.

da Cunha MG, de Cássia Orlandi Sardi J, Freires IA, Franchin M, Rosalen PL. Antimicrobial, anti-adherence and antibiofilm activity against Staphylococcus aureus of a 4-phenyl coumarin derivative isolated from Brazilian geopropolis. Microb Pathogen. 2020;139:103855.

Ekambaram SP, Perumal SS, Balakrishnan A, Marappan N, Gajendran SS, Viswanathan V. Antibacterial synergy between rosmarinic acid and antibiotics against methicillin-resistant Staphylococcus aureus. J Intercult Ethnopharmacol. 2016;5(4):358–363.

Gibbons S, Leimkugel J, Oluwatuyi M, Heinrich M. Activity of Zanthoxylum clava-herculis extracts against multi-drug resistant methicillin-resistant Staphylococcus aureus (mdr-MRSA). Phytother Res. 2003;17(3):274–275.

Gupta VK, Tiwari N, Gupta P, Verma S, Pal A, Srivastava SK, et al. A clerodane diterpene from Polyalthia longifolia as a modifying agent of the resistance of methicillin resistant Staphylococcus aureus. Phytomedicine. 2016;23(6):654–661.

Hatano T, Kusuda M, Hori M, Shiota S, Tsuchiya T, Yoshida T. Theasinensin A, a tea polyphenol formed from (-)-epigallocatechin gallate, suppresses antibiotic resistance of methicillin-resistant Staphylococcus aureus. Planta Med. 2003;69(11):984–989.

Joung DK, Joung H, Yang DW, Kwon DY, Choi JG, Woo S, et al. Synergistic effect of rhein in combination with ampicillin or oxacillin against methicillin-resistant Staphylococcus aureus. Exp Ther Med. 2012; 3(4):608–612.

Joung DK, Lee YS, Han SH, Lee SW, Cha SW, Mun SH, et al. Potentiating activity of luteolin on membrane permeabilizing agent and ATPase inhibitor against methicillin-resistant Staphylococcus aureus. Asian Pac J Trop Med. 2016a;9(1):19–22.

Joung DK, Mun SH, Choi SH, Kang OH, Kim SB, Lee YS, et al. Antibacterial activity of oxyresveratrol against methicillin-resistant Staphylococcus aureus and its mechanism. Exp Ther Med. 2016b;12(3):1579–1584.

Kang HK, Kim HY, Cha JD. Synergistic effects between silibinin and antibiotics on methicillin-resistant Staphylococcus aureus isolated from clinical specimens. Biotechnol J. 2011;6(11):1397–1408.

Kumarihamy M, Tripathi SK, Khan S, Muhammad I. Schottiin, a new prenylated isoflavones from Psorothamnus schottii and antibacterial synergism studies between methicillin and fremontone against methicillin-resistant Staphylococcus aureus ATCC 1708. Nat Prod Res. 2022;36(12):2984–2992.

Lan JE, Li XJ, Zhu XF, Sun ZL, He JM, Zloh M, et al. Flavonoids from Artemisia rupestris and their synergistic antibacterial effects on drug-resistant Staphylococcus aureus. Nat Prod Res. 2021;35(11):1881–1886.

Lee YS, Kang OH, Choi JG, Oh YC, Chae HS, Kim JH, et al. Synergistic effects of the combination of galangin with gentamicin against methicillin-resistant Staphylococcus aureus. J Microbiol. 2008;46:283–288.

Lee YS, Lee DY, Kim YB, Lee SW, Cha SW, Park HW, et al. The mechanism underlying the antibacterial activity of shikonin against methicillin-resistant Staphylococcus aureus. Evid Based Complement Altern Med. 2015;2015:520578.

Liu IX, Durham DG, Richards RM. Baicalin synergy with β-lactam antibiotics against methicillin-resistant Staphylococcus aureus and other β-lactam-resistant strains of S. aureus. J Pharm Pharmacol. 2000;52 (3):361–366.

Merghni A, Noumi E, Hadded O, Dridi N, Panwar H, Ceylan O, et al. Assessment of the antibiofilm and antiquorum sensing activities of Eucalyptus globulus essential oil and its main component 1,8-cineole against methicillin-resistant Staphylococcus aureus strains. Microb Pathog. 2018;118:74–80.

Mohtar M, Johari SA, Li AR, Isa MM, Mustafa S, Ali AM, et al. Inhibitory and resistance-modifying potential of plant-based alkaloids against methicillin-resistant Staphylococcus aureus (MRSA). Curr Microbiol. 2009;59:181–186.

Okamura S, Nishiyama E, Yamazaki T, Otsuka N, Taniguchi S, Ogawa W, et al. Action mechanism of 6,6′-dihydroxythiobinupharidine from Nuphar japonicum, which showed anti-MRSA and anti-VRE activities. Biochim Biophys Acta. 2015;1850(6):1245–1252.

Sakagami Y, Mimura M, Kajimura K, Yokoyama H, Iinuma M, Tanaka T, et al. Anti-MRSA activity of sophoraflavanone G and synergism with other antibacterial agents. Lett Appl Microbiol. 1998;27(2):98–100.

Septama AW, Panichayupakaranant P. Synergistic effect of artocarpin on antibacterial activity of some antibiotics against methicillin-resistant Staphylococcus aureus, Pseudomonas aeruginosa, and Escherichia coli. Pharm Biol. 2016;54(4):686–691.

Sharifi-Rad J, Hoseini-Alfatemi S, Sharifi-Rad M, Iriti M. Antimicrobial synergic effect of Allicin and silver nanoparticles on skin infection caused by methicillin resistant Staphylococcus aureus spp. Ann Med Health Sci Res. 2014;4(6):863–868.

Tan S, Hua X, Xue Z, Ma J. Cajanin stilbene acid inhibited vancomycin-resistant Enterococcus by inhibiting phosphotransferase system. Front Pharmacol. 2020;11:473.

Vázquez NM, Fiorilli G, Guido PAC, Moreno S. Carnosic acid acts synergistically with gentamicin in killing methicillin-resistant Staphylococcus aureus clinical isolates. Phytomedicine. 2016;23(12):1337–1343.

Wang F, Liu H, Li J, Zhang W, Jiang B, Xuan H. Australian propolis ethanol extract exerts antibacterial activity against methicillin-resistant Staphylococcus aureus by mechanisms of disrupting cell structure, reversing resistance, and resisting biofilm. Braz J Microbiol. 2021;52:1651–1664.

Yu HH, Kim KJ, Cha JD, Kim HK, Lee YE, Choi NY, et al. Antimicrobial activity of berberine alone and in combination with ampicillin or oxacillin against methicillin-resistant Staphylococcus aureus. J Med Food. 2005;8 (4): 454–461.

Zajmi A, Mohd Hashim N, Noordin MI, Khalifa SA, Ramli F, Mohd Ali H, et al. Ultrastructural study on the antibacterial activity of artonin e versus streptomycin against Staphylococcus aureus strains. PLoS One. 2015;10(6):e0128157.

Zhang S, Hu B, Xu J, Ren Q, Wang Z, Wang S, et al. Baicalin suppress growth and virulence-related factors of methicillin-resistant Staphylococcus aureus in vitro and vivo. Microb Pathog. 2020;139:103899.

Zhou T, Li Z, Kang OH, Mun SH, Seo YS, Kong R, et al. Antimicrobial activity and synergism of ursolic acid 3-O-α-L-arabinopyranoside with oxacillin against methicillin-resistant Staphylococcus aureus. Int J Mol Med. 2017;40(4):1285–1293.

Zuo G, Han Z, Hao X, Han J, Li Z, Wang GC. Synergy of aminoglycoside antibiotics by 3-Benzylchroman derivatives from Caesalpinia sappan against clinical methicillin-resistant Staphylococcus aureus (MRSA). Phytomedicine. 2014;21(7):936–941.
